# Burden and risk factors of sexually transmitted infections before and after HIV diagnosis in a Finnish national HIV cohort, 1995–2019

**DOI:** 10.1017/S0950268826101150

**Published:** 2026-02-16

**Authors:** Aurora Kaila, Inka Aho, Kirsi Liitsola, Eija Hiltunen-Back, Henrikki Brummer-Korvenkontio, Jukka Ollgren, Pia Kivelä

**Affiliations:** 1 Faculty of Medicine, University of Helsinki, Helsinki, Finland; 2 Department of Public Health, Finnish Institute for Health and Welfare, Helsinki, Finland; 3 Department of Infectious Diseases, Inflammation Centre, Helsinki University Hospital, Helsinki, Finland; 4 Venereal Diseases Outpatient Clinic, Inflammation Centre, Helsinki University Hospital, Helsinki, Finland

**Keywords:** HIV, sexually transmitted infections, HIV testing, indicator conditions, PrEP

## Abstract

Sexually transmitted infections (STIs) are common among people living with human immunodeficiency virus (HIV) (PLWH). This nationwide register study linked HIV and STI registries to examine STI trends before and after HIV diagnosis in Finland 1995–2019 among all PLWH residing in the country. Analysed STIs were chlamydia, gonorrhoea, syphilis, and hepatitis B and C. An extended Cox model assessed factors associated with STI events. Among 3,775 PLWH (mean follow-up 17.9 person-years), 71% had no STIs, 17% had one, and 12% had two or more. Overall, 10.7% had an STI before HIV diagnosis and 18.1% after. STI incidence was 32 per 1,000 person-years and increased over time, although chlamydia and gonorrhoea declined. STI risk was highest among men who have sex with men (MSM) and lowest among people who inject drugs; it remained stable or declined after HIV diagnosis. STIs before HIV diagnosis offer opportunities for HIV testing and pre-exposure prophylaxis promotion. As most had no STIs other than HIV, HIV testing should not be limited to STI screening but also performed in other indicator conditions. After HIV diagnosis, accessible low-threshold STI testing, particularly for MSM, and consideration of doxycycline prophylaxis may benefit those at highest risk.

## Introduction

Early diagnosis of human immunodeficiency virus (HIV) and prompt initiation of antiretroviral therapy (ART) are crucial both for improving individual prognosis and for preventing onward transmission of HIV, thereby contributing to the goal of ending the HIV pandemic [1]. When the viral load is low, HIV is not transmitted through condomless sex – reflected in the principle ‘undetectable = untransmittable’ (U=U) [2–5]. However, other sexually transmitted infections (STIs) continue to be transmitted and are increasing in incidence among people living with HIV (PLWH) [6–9]. If left untreated, some STIs can cause serious harm. Moreover, they increase the risk of both acquiring and transmitting HIV through mechanisms such as genital ulceration and mucosal inflammation [10].

HIV prevalence remains low in Finland. Since the 2000s, approximately 150 new HIV cases have been reported annually, corresponding to an incidence of 2.4–3.6 per 100,000 population. Under Finnish law, testing, treatment, and medication for HIV, as well as for chlamydia, syphilis, and gonorrhoea, are provided free of charge. In Finland, HIV care is provided exclusively in public universities and central hospitals. Typically, patients have two contacts with the HIV clinic per year, with at least one being an in-person visit. STI tests are not part of routine examinations during these contacts, except for syphilis serology, which is performed annually in most clinics. Medications for hepatitis B and C are also available at no cost. All these infections are notifiable by law and must be recorded in the National Infectious Diseases Register (NIDR). HIV testing is routinely recommended whenever an STI is diagnosed or suspected [11]. Free HIV testing, available without referral and anonymously upon request, is widely accessible to everyone at health centres, STI outpatient clinics, student, and occupational health services and through non-governmental providers. However, undocumented migrants may face barriers to services due to language and access issues. Since 1998, opt-out antenatal screening for HIV, syphilis, and hepatitis B has been part of routine maternal care. People who inject drugs (PWID), men who have sex with men (MSM), and sex workers are eligible for free hepatitis B vaccination, although data on vaccination coverage are not available. HIV pre-exposure prophylaxis (HIV PrEP) has been available in Finland since 2019 and has been provided free of charge within the public healthcare system since July 2021. As of 2023, Finland has met the Joint United Nations Programme for HIV/acquired immunodeficiency syndrome (AIDS) (UNAIDS) 95–95–95 targets for HIV testing, treatment, and viral suppression, originally set for achievement by 2025 [12].

At the national level, data on the impact of HIV diagnosis on subsequent STI incidence remain limited. Similarly, long-term follow-up data on how the U=U era has influenced the STI incidence among PLWH are scarce. This national registry-based study examines STI incidence among all Finnish adult PLWH over a 25-year period, providing indirect insights into behavioural changes before and after HIV diagnosis. The analysed STIs included chlamydia (with a few cases of lymphogranuloma venereum), as well as syphilis, gonorrhoea, and hepatitis B and C. The primary objective is to identify populations at increased risk who may benefit from targeted interventions to reduce STI transmission.

## Methods

### Data sources and study population

This nationwide retrospective register-based study integrates data from two national registers: the NIDR and the Finnish HIV Quality of Care Register (FINHIV). The NIDR, maintained by the Finnish Institute for Health and Welfare (THL) since 1995, collects mandatory notifications of communicable diseases from both laboratories and physicians. The laboratory notifications are automated, and together with physician notifications, they provide a highly comprehensive dataset. The FINHIV includes epidemiological and treatment outcome data for all individuals diagnosed with or treated for HIV in Finland [13]. Data from the two registers were linked using Finland’s unique 10-digit personal identity codes (PICs), which are assigned to all citizens at birth and to foreign residents who are registered in the Population Information System. PICs enable accurate identification of individuals across official records and health registers. The linkage of the registers produced a comprehensive national dataset covering all individuals diagnosed or treated for HIV in Finland since 1984, along with their notifiable STIs recorded since 1995. The analysis covered the period from 1 January 1995 to 31 December 2019, beginning with the availability of electronic STI data after the NIDR was established.

### Inclusion criteria

The inclusion criteria were age ≥ 16 years at the start of follow-up, with participants required to reach age 18 by the end of 2019, and successful data linkage between the registers.

Individual follow-up periods were defined separately for each person, both before and after HIV diagnosis. For individuals of Finnish origin, the start of the diagnostic follow-up period was set as either 1 January 1995 or the date they turned 16, whichever occurred later. For individuals of non-Finnish origin, additional criteria – such as the date of obtaining a residence permit, municipal registration, or issuance of a PIC – were used to refine the start of follow-up, adjusting for the pre-admission period. If a reliable start date could not be determined after these adjustments, follow-up was defined as beginning on the date of HIV diagnosis or the first recorded STI, whichever came first. The end of the diagnostic follow-up period was defined as the date of death, emigration, or 31 December 2019, whichever occurred first. For individuals of non-Finnish origin, 31 December 2019 was only accepted as the cut-off date if an HIV viral load measurement were available for the year 2019. In the absence of viral load data between 2017 and 2019, individuals were considered loss to follow-up or likely to have emigrated. In these cases, follow-up was censored at the latest of the following events: last treatment contact, last HIV viral load measurement, last STI diagnosis, or initiation of ART.

### Definitions

A single STI diagnostic event could include one or more concurrent cases, meaning simultaneous diagnoses of different STIs. The STIs analysed were chlamydia, syphilis, gonorrhoea, and hepatitis B and C. In the NIDR, multiple episodes of chlamydia and gonorrhoea in the same individual within a 90-day period are recorded as a single case, while syphilis and hepatitis B and C are counted only once per person over a 50-year period. For this study, we refined the recurrence interval for syphilis as 13 months to better reflect clinical reinfection, while the original recurrence intervals for hepatitis B and C remained unchanged. In this study, we defined hepatitis B and C infections among PWID as injection-related rather than sexually transmitted, and the juvenile hepatitis B case was assumed to be congenital. STIs diagnosed concurrently with HIV were classified as having occurred prior to HIV.

### Statistical analysis

Data preparation, descriptive statistics on cohort demographics, and STI frequency analyses were conducted using Statistical Package for the Social Sciences (SPSS) 25.0 (IBM, Chicago, Illinois, USA). Time-dependent analyses were performed using Stata 18.0 (Stata Corp LLC, College Station, Texas, USA).

Incidence rates of specific STI pathogens before and after HIV diagnosis were calculated based on STI diagnostic events rather than individual cases.

Predicted margins of hazard ratios for STI diagnostic events were calculated using an extended Cox proportional hazards model, specifically the Andersen–Gill formulation. The model estimates hazard ratios for each group relative to a common baseline, with all covariates set to zero. The model allows direct comparison of multiple groups by including categorical covariates in the regression. Each group’s effect is quantified as a hazard ratio, interpreted as the relative event rate compared to the baseline. In this model, age was used as the primary time scale rather than included as a covariate. The model incorporated several covariates, including time periods relative to HIV diagnosis (before vs. after), HIV transmission mode, calendar time intervals (treated as categorical variables), hospital district, gender, origin (Finnish or non-Finnish), and interactions among these variables. Interactions were specifically tested between the following pairs: time period relative to HIV diagnosis and HIV transmission mode; time period relative to HIV diagnosis and calendar time interval, HIV transmission mode and calendar time interval, gender as a main effect; hospital district and HIV transmission mode; and hospital district and origin.

Analyses were conducted for the calendar periods 1995–1999, 2000–2003, 2004–2007, 2008–2011, and 2012–2015, as well as the shorter periods 2016–2018 and 2019. The year 2019 was analysed separately due to the small number of individuals contributing follow-up time before their HIV diagnosis. We selected approximately equal intervals, considering major milestones in HIV treatment guidelines, such as the 2008 Swiss National HIV/AIDS Commission (the ‘Swiss Statement’) [2] and the 2016 Partners of People on ART—A New Evaluation of the Risks (PARTNER1) study [3], which showed that PLWH with sustained viral suppression do not transmit HIV. These milestones provide context for our indirect assessments of potential behavioural changes among PLWH.

To evaluate the robustness of the findings, we conducted multiple sensitivity analyses. First, the model was re-run excluding STIs diagnosed in the month prior to HIV diagnosis, to control for potential bias introduced by simultaneous HIV and STI testing. Additional sensitivity analyses restricted the follow-up periods to two and three years before and after HIV diagnosis, aiming to reduce bias related to unequal observation periods in the pre- and post-diagnosis phases. Finally, we evaluated the impact of excluding all individuals without pre-HIV follow-up to assess whether their inclusion influenced the main results.

In Finland, retrospective register-based studies do not require approval from an ethics committee or informed consent from participants. This study was approved by the THL in 2019 (THL/1572/6.02.00/2019).

## Results

### Study population

The dataset initially comprised all PLWH living in Finland during the study period, whether diagnosed before or during the study. A total of 470 individuals – primarily immigrants with temporary residence permits – were excluded because of inconsistent PICs, most often due to multiple moves to or from Finland. In addition, 67 individuals without recorded viral load data between 2017 and 2019 were classified as lost to follow-up or presumed to have emigrated. The final dataset included 3,775 PLWH, the majority of whom were male, of Finnish origin, and residing in the Helsinki University Hospital area. The median cluster of differentiation 4 (CD4) count at HIV diagnosis was 393 cells/μL, and the mean overall follow-up per participant was 17.9 person-years (6.9 before and 11.0 after HIV diagnosis). Table 1 summarizes the study population.

**Table 1.** Characteristics of the Finnish HIV Cohort, 1995–2019

	*n* = 3,775 (%) (100.0%)^a^	No STI (*n* = 2,690) (%) (71.3)^a^	≥1 STI (*n* = 1,085) (%) (28.7)^a^
Age at HIV diagnosis (years)			
Median (range)	36.5 (16–81)	37.3 (16–81)	33.7 (16–72)
Gender^b^			
Men	2,658 (70.4)	1796 (66.8)	862 (79.4)
Women	1,071 (28.4)	865 (32.2)	206 (19.0)
Missing	46 (1.2)	29 (1.1)	17 (1.6)
Origin			
Finnish	2,442 (64.7)	1709 (63.5)	733 (67.6)
Other	1,162 (30.8)	836 (31.1)	326 (30.0)
Missing	171 (4.5)	145 (5.4)	26 (2.4)
Hospital district			
HUS^c^	2,254 (59.7)	1,555 (57.8)	699 (64.4)
Other	1,475 (39.1)	1,106 (41.1)	369 (34.0)
Missing	46 (1.2)	29 (1.1)	17 (1.6)
HIV transmission mode			
Heterosexual contact	1738 (46.0)	1,330 (49.4)	408 (37.6)
Male-to-male sexual contact	1,216 (32.2)	645 (24.0)	571 (52.6)
Injecting drug use	413 (10.9)	390 (14.5)	23 (2.1)
Other or not known	408 (10.8)	325 (12.1)	83 (7.6)
Year of HIV diagnosis			
1980–1989	190 (5.0)	167 (6.2)	23 (2.1)
1990–1999	677 (17.9)	546 (20.3)	131 (12.1)
2000–2009	1,500 (39.7)	1,053 (39.1)	447 (41.2)
2010–2019	1,362 (36.1)	895 (33.3)	467 (43.0)
Missing	46 (1.2)	29 (1.1)	17 (1.6)
First CD4 count (cells/μL) range: 0–2000			
<200	816 (21.6)	605 (22.5)	211 (19.4)
201–350	668 (17.7)	459 (17.1)	209 (19.3)
>351	1910 (50.6)	1,291 (48.0)	619 (57.1)
Missing	381 (10.1)	335 (12.5)	46 (4.2)

a Row percentage.

b The register does not identify other genders.

c Helsinki University Hospital, capital area of Finland.

### Person-years of follow-up

The study encompassed 25,909 person-years of follow-up (PYFU) before HIV diagnosis and 41,847 PYFU after HIV diagnosis. Before HIV diagnosis, 45.5% of follow-up time was accumulated by heterosexual individuals, 37.3% by MSM, 8.3% by PWID, and 8.8% by those with other or unknown transmission modes. After HIV diagnosis, the corresponding proportions were 44.2%, 36.0%, 12.2%, and 7.5%, respectively.

### STI occurrence before and after HIV diagnosis

**Figure 1.**
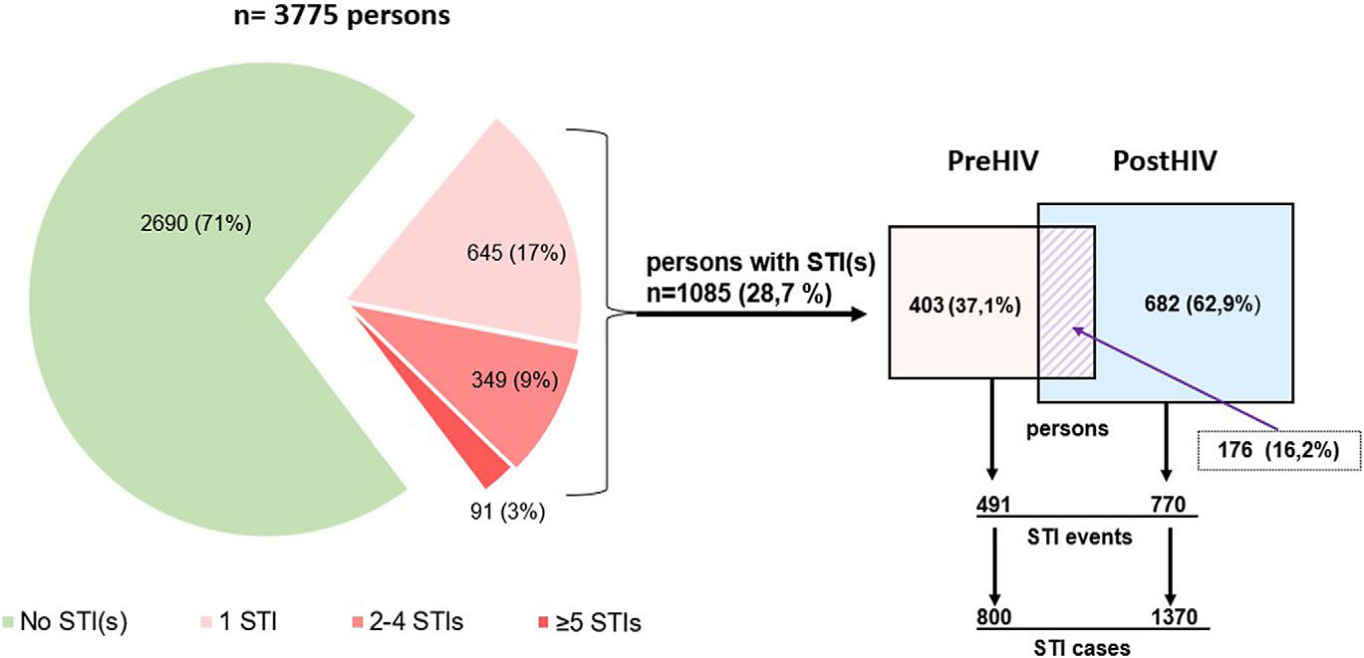
Presence of sexually transmitted infections and their temporal relationship to HIV diagnosis in the Finnish HIV Cohort, 1995–2019. *Note:* 10.7 % had an STI prior to HIV diagnosis and 18.1 % after it.

Over the 25-year observation period, 2,690 (71.3%) people had no recorded STIs other than HIV, while 1,085 individuals (28.7%) had at least one STI. Among those with any STI, 59.4% (*n* = 645) had one case, 32.2% (*n* = 349) had two to four cases, and 8.4% (*n* = 91) had five or more cases. The proportion of individuals with no recorded STIs other than HIV was 76.5% among heterosexual individuals and 53.0% among MSM. Overall, only 10.7% of individuals in the entire cohort had an STI prior to their HIV diagnosis and 18.1% after the HIV diagnosis. (Figure 1).

A total of 2,170 STI cases were identified across 1,261 distinct diagnostic events. Of the 2,170 STI cases, 800 (36.9%) occurred before the HIV diagnosis and 1,370 (63.1%) after it. The corresponding numbers of diagnostic events were 491 (39.9%) in the pre-HIV period and 770 (61.1%) in the post-HIV period (Figure 1).

**Figure 2.**
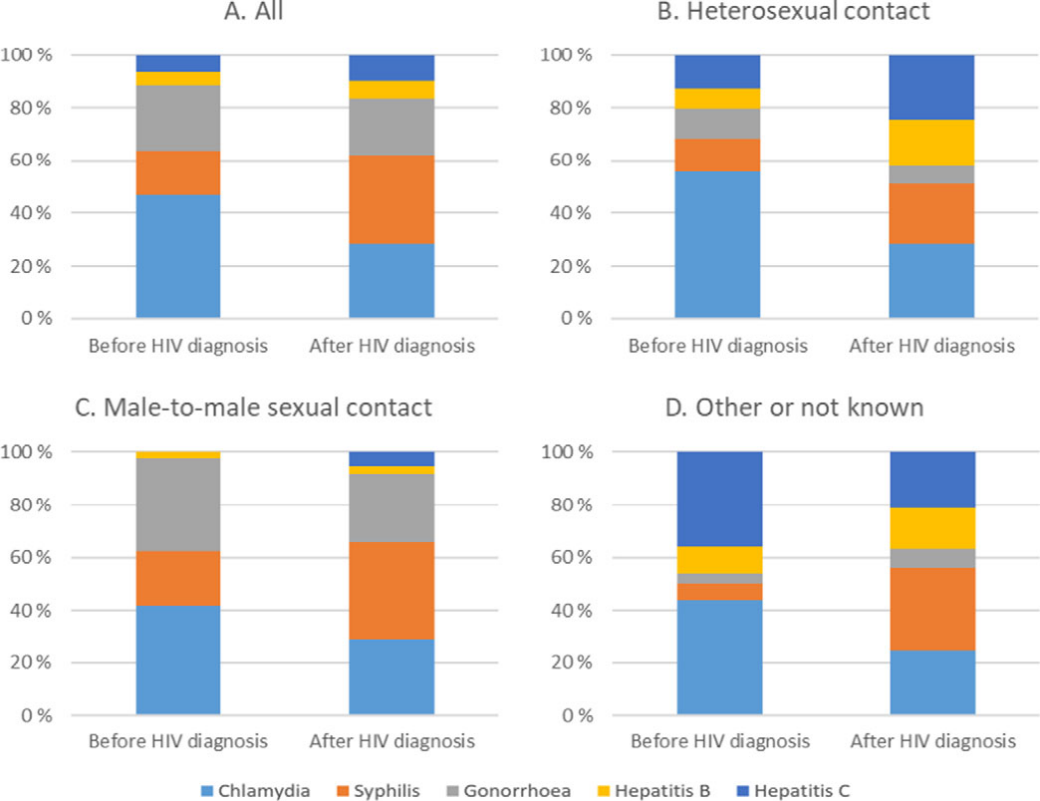
Percentage distribution of sexually transmitted infection cases (n = 2170) by HIV transmission mode before and after HIV diagnosis in the Finnish HIV Cohort, 1995–2019.

The number of positive diagnostic events per person ranged from 1 to 20, with a median of 1. The number of STI cases per person ranged from 1 to 28, also with a median of 1. Chlamydia was the most common STI overall (*n* = 794, 36.6%) and across all HIV transmission modes. It was also the most common STI before HIV diagnosis (*n* = 374, 46.7%). After HIV diagnosis, syphilis became the most frequent STI (*n* = 460, 33.6%) (Table 2, Figure 2).

**Table 2.** STI diagnostic events and cases by HIV transmission modes in the Finnish HIV Cohort, 1995–2019

	Heterosexual contact (*n* (%))	Male-to-male sexual contact (*n* (%))	Injecting drug use (*n* (%))	Other or not known (*n* (%))	Total (*n* (%))
STI events	443 (35.1)^a^	708 (56.1)^a^	25 (2.0)^a^	85 (67.0)^a^	1,261 100.0^a^
1–20/person median 1					
1	362 (81.7)	374 (52.8)	21 (84.0)	73 (85.9)	830 (65.8)
2–4	77 (17.4)	275 (38.8)	4 (16.0)	12 (14.1)	368 (29.2)
≥ 5	4 (0.9)	59 (8.3)	0 (0)	0 (0)	63 (5.0)
STI cases	551	1,491	30	98	2,170
1–28/person median 1					
1	313 (76.7)	244 (42.7)	18 (78.3)	70 (84.3)	645 (29.7)
2–4	89 (21.8)	242 (42.4)	5 (21.7)	13 (15.7)	349 (18.2)
≥ 5	6 (1.5)	85 (14.9)	0 (0)	0 (0)	91 (4.2)
Chlamydia	224 (40.7)	521 (34.9)	22 (73.3)	27 (27.6)	794 (36.6)
Syphilis	100 (18.1)	474 (31.8)	7 (23.3)	16 (16.3)	597 (27.5)
Gonorrhoea	50 (9.1)	401 (26.9)	1 (3.3)	8 (8.2)	460 (21.2)
Hepatitis B	72 (13.1)	42 (2.8)	N/A^b^	15 (15.3)	129 (5.9)
Hepatitis C	105 (19.1)	53 (3.6)	N/A^b^	32 (32.7)	190 (8.8)

*Note*: An STI diagnostic event refers to a diagnostic encounter during which one or more STI cases may be identified concurrently. A single individual may experience multiple STI diagnostic events over the follow-up period, with one or more STI cases diagnosed during each diagnostic event. This table presents the number of diagnostic events and cases aggregated over the entire study period.

a Row percentage.

b N/A = not applicable. HBV (*n* = 60) and HCV (*n* = 282) are not counted as STIs among PWID.

Although MSM represented only 32.6% of the study population, they accounted for 56.1% (*n* = 708) of all STI diagnostic events and 68.7% (*n* = 1,491) of all STI cases, whereas heterosexuals accounted for 25,4% (*n* = 551) and PWID for 1,4% (*n* = 30). MSM also accounted for most of the chlamydia, syphilis, and gonorrhoea cases. Figure 2 illustrates the percentage distribution of chlamydia, gonorrhoea, syphilis, and hepatitis B and C before and after HIV diagnosis, stratified by HIV transmission group.

### STI incidence trends

**Figure 3.**
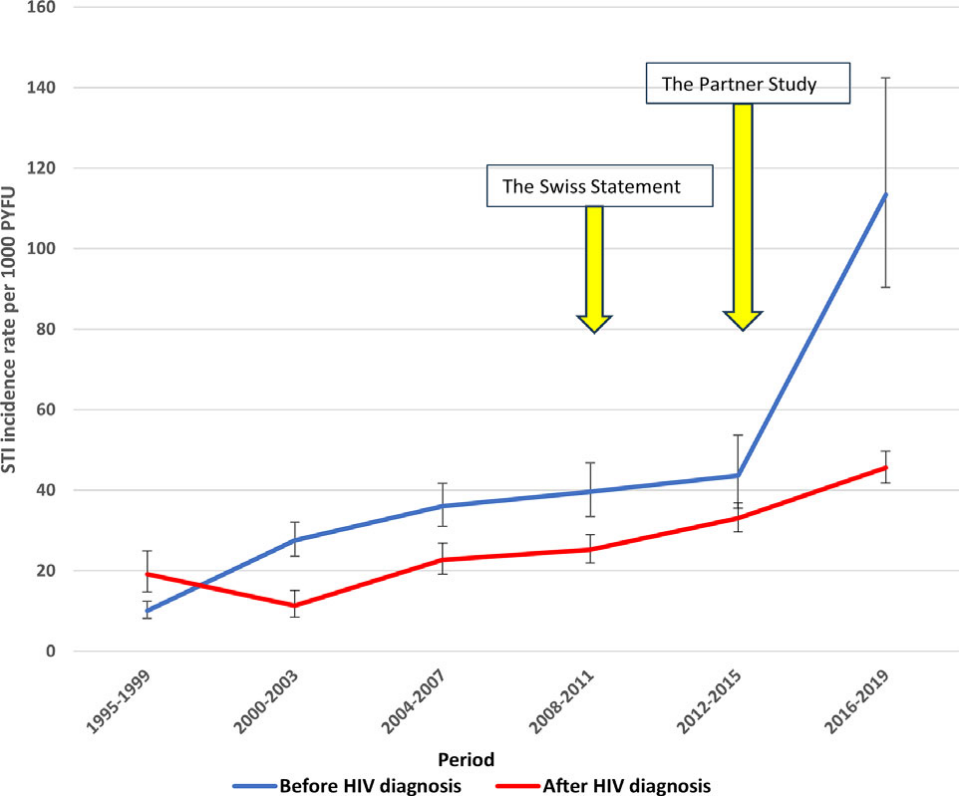
Sexually transmitted infection event incidence rates before and after HIV diagnosis in the Finnish HIV Cohort, 1995–2019. *Note:* The yellow arrows indicate the publication or dissemination of data demonstrating that well-treated HIV infection is untransmittable. The first results of the PARTNER study were reported in 2014 and published in 2016.

Over the 25-year follow-up period, the overall incidence rate of STI diagnostic events was 32.1 per 1,000 PYFU (95 per cent confidence interval (95% CI), 30.8–33.5). This rate increased slightly from 31.0/1000 PYFU (95% CI, 28.9–33.2) before HIV diagnosis to 32.8/1000 PYFU (95% CI, 31.1–34.6) after HIV diagnosis, based on diagnostic event counts within defined time intervals. After the first period (1995–1999), the annual incidence rate of STI diagnostic events within a period was consistently higher before HIV diagnosis than after HIV. No statistically significant increase in STI incidence was observed immediately following the introduction of the Swiss statement [2]. However, after the 2012–2015 follow-up period, STI incidence rates increased significantly both before and after HIV diagnosis (Figure 3).

When comparing STI incidence across HIV transmission modes and time periods before and after HIV diagnosis, the highest incidence was observed among MSM after HIV diagnosis: 61.9/1000 PYFU (95% CI, 58.0–66.0). Among specific infections, chlamydia had the highest overall incidence and was most common before HIV diagnosis. After HIV diagnosis, syphilis became the most frequent STI, whereas chlamydia and gonorrhoea incidences declined when assessed individually. (Table 3).

**Table 3.** Sexually transmitted infection incidences before and after HIV diagnosis in the Finnish HIV cohort, 1995–2019

	STI event incidences/1000 PYFU (95% CI)		
	Before HIV	After HIV	Total
All	31.0 (28.9–33.2)	32.8 (31.1–34.6)	32.1 (30.8–33.5)
Gender			
• Men	29.6 (27.3–32.0)	37.6 (35.5–39.9)	34.3 (32.7–35.9)
• Women	22.6 (18.7–27.3)	12.7 (10.8–14.9)	15.5 (13.7–17.5)
Origin			
• Finnish	26.7 (24.7–28.9)	29.9 (28.0–31.9)	28.5 (27.1–30.0)
• Other	43.3 (35.9–52.2)	34.0 (30.6–37.8)	35.9 (32.7–39.3)
Hospital district			
• HUS^a^	35.2 (32.2–38.3)	35.4 (33.2–37.7)	35.3 (33.5–37.2)
• Other	19.6 (17.2–22.3)	21.7 (19.4–24.1)	20.8 (19.1–22.6)
HIV transmission mode			
• Heterosexual contact	22.0 (19.5–24.9)	15.4 (13.7–17.2)	18.0 (16.5–19.5)
• Male-to-male sexual contact	43.6 (39.7–48.0)	61.9 (58.0–66.0)	54.7 (51.9–57.7)
• Injecting drug use	4.6 (2.5–8.6)	3.9 (2.5–6.1)	4.1 (2.9–5.9)
• Other or not known	17.6 (12.9–24.0)	11.8 (8.5–16.3)	14.2 (11.4–17.8)

	STI case incidences/1000 PYFU (95% CI)		
	Before HIV	After HIV	Total
• Chlamydia	14.3 (13.0–15.9)	9.4 (8.5–10.3)	11.7 (10.9–12.6)
• Syphilis	5.3 (4.5–6.3)	11.0 (10.1–12.1)	8.83 (8.15–9.56)
• Gonorrhoea	7.7 (6.7–8.8)	6.3 (5.6–7.1)	6.80 (6.20–7.45)
• Hepatitis B	1.4 (1.0–2.0)	2.2 (1.8–2.7)	1.89 (1.59–2.25)
• Hepatitis C	2.0 (1.5–2.6)	3.3 (2.8–3.9)	2.81 (2.44–3.24)

*Note*: Incidence rates calculated based on diagnostic events per defined time intervals.

a Helsinki University Hospital, capital area of Finland.

### Regression and sensitivity analyses

**Figure 4.**
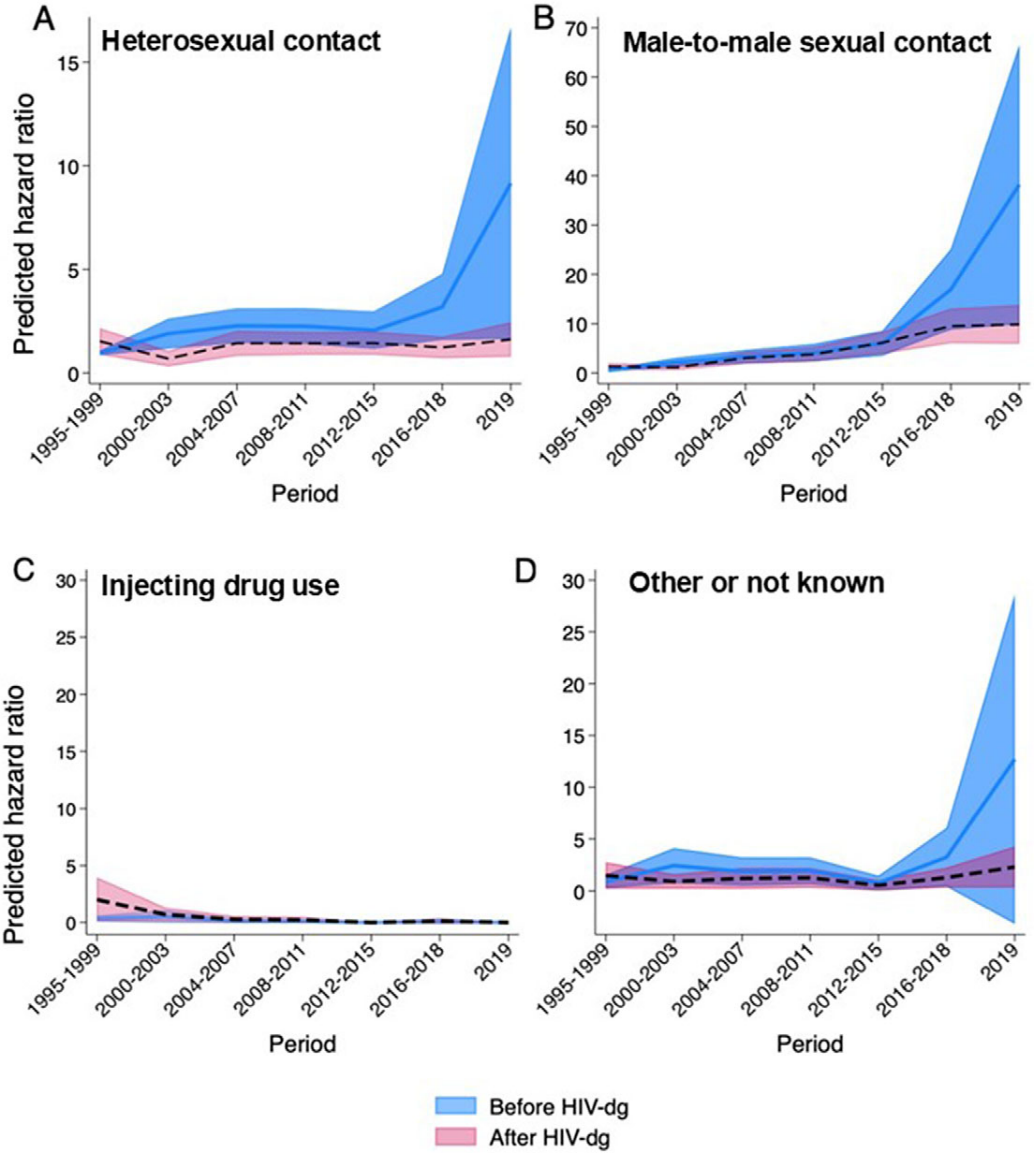
Predicted margins for hazard ratios for sexually transmitted infections by HIV transmission mode in the Finnish HIV Cohort, 1995–2019 (95% confidence intervals). *Note:* The y-axis ranges from 015 in panel A, from 070 in panel B, and from 030 in panels C and D. The same baseline reference category is used across all panels.

The extended Cox model showed that the predicted margins for hazard ratios of STIs after an HIV diagnosis were the same as or lower than before the HIV diagnosis, regardless of HIV transmission mode. Among MSM and heterosexual transmission groups, the predicted margins for the hazard ratio before HIV diagnosis appear to increase from the period 2012–2015 onwards, but the difference is not statistically significant (Figure 4 and Table 4). When STIs diagnosed in the month preceding the HIV diagnosis were excluded from the analysis, the adjusted predicted margins for hazard ratios among heterosexuals and MSM in 2019 were nearly halved. However, the risk remained higher than in the earlier study period (Supplementary Figure 1 and Supplementary Table 1). Sensitivity analyses limited to follow-up periods of two and three years before and after HIV diagnosis revealed no evidence of differences in predictive margins for hazard ratios for STIs between the pre- and post-HIV periods.

**Table 4.** Predicted margins for hazard ratios for sexually transmitted infections by HIV transmission modes in the Finnish HIV Cohort, 1995–2019

All HIV transmission modes							
Time period	Relation to HIV diagnosis	Margin	Delta method std. err.	*z*	*P* > |*z*|	95% CI	
1995–1999	Before	0.743373	0.0838473	8.87	0.000	0.5790353	0.9077107
	After	1.490114	0.3238547	4.60	0.000	0.8553706	2.124858
2000–2003	Before	1.931927	0.3563268	5.42	0.000	1.233539	2.630314
	After	0.900286	0.2086124	4.32	0.000	0.4914133	1.309159
2004–2007	Before	2.485484	0.4488994	5.54	0.000	1.605657	3.365311
	After	1.976231	0.3729307	5.30	0.000	1.2453	2.707161
2008–2011	Before	2.807389	0.5371882	5.23	0.000	1.754519	3.860258
	After	2.281218	0.4085938	5.58	0.000	1.480389	3.082047
2012–2015	Before	3.420187	0.684544	5.00	0.000	2.078505	4.761868
	After	3.172226	0.5648658	5.62	0.000	2.06511	4.279343
2016–2018	Before	8.571231	1.992186	4.30	0.000	4.666618	12.47584
	After	4.549486	0.8251868	5.51	0.000	2.93215	6.166822
2019	Before	20.51133	7.6076	2.70	0.007	5.600704	35.42195
	After	4.907612	0.9537664	5.15	0.000	3.038264	6.776959
Heterosexual contact							
1995–1999	Before	0.9639655	0.0521393	18.49	0.000	0.8617743	1.066157
	After	1.528307	0.3280489	4.66	0.000	0.8853428	2.171271
2000–2003	Before	1.903293	0.3688348	5.16	0.000	1.18039	2.626196
	After	0.6907244	0.1902234	3.63	0.000	0.3178933	1.063555
2004–2007	Before	2.283716	0.4276843	5.34	0.000	1.44547	3.121962
	After	1.434086	0.3110568	4.61	0.000	0.8244256	2.043746
2008–2011	Before	2.26471	0.4396858	5.15	0.000	1.402941	3.126478
	After	1.429175	0.2820255	5.07	0.000	0.8764153	1.981935
2012–2015	Before	2.071003	0.4583264	4.52	0.000	1.1727	2.969306
	After	1.43927	0.2857191	5.04	0.000	0.8792711	1.99927
2016–2018	Before	3.189211	0.8083087	3.95	0.000	1.604955	4.773467
	After	1.227994	0.2636558	4.66	0.000	0.7112385	1.74475
2019	Before	9.156147	3.829866	2.39	0.017	1.649748	16.66255
	After	1.61776	0.4253234	3.80	0.000	0.7841417	2.451379
Male-to-male sexual contact							
1995–1999	Before	0.5645711	0.1294291	4.36	0.000	0.3108946	0.8182476
	After	1.308514	0.3492792	3.75	0.000	0.6239391	1.993088
2000–2003	Before	2.173079	0.4570861	4.75	0.000	1.277207	3.068951
	After	1.152883	0.2881254	4.00	0.000	0.5881674	1.717598
2004–2007	Before	3.311785	0.6731559	4.92	0.000	1.992424	4.631147
	After	3.040225	0.6055435	5.02	0.000	1.853382	4.227068
2008–2011	Before	4.107144	0.8926947	4.60	0.000	2.357495	5.856794
	After	3.78899	0.7298985	5.19	0.000	2.358415	5.219565
2012–2015	Before	6.028156	1.290749	4.67	0.000	3.498334	8.557977
	After	6.124304	1.136545	5.39	0.000	3.896716	8.351892
2016–2018	Before	16.95676	4.140539	4.10	0.000	8.841452	25.07207
	After	9.544792	1.797768	5.31	0.000	6.021231	13.06835
2019	Before	38.1864	14.41096	2.65	0.008	9.94143	66.43136
	After	9.863264	2.016941	4.89	0.000	5.910132	13.8164

*Note*: The same baseline reference category is used accross all panels.

A total of 1,154 individuals were missing pre-HIV follow-up time due to reasons such as an HIV diagnosis before 1995 (*n* = 440; 389 Finnish and 51 foreign-born), entry into the study cohort after HIV diagnosis – often due to immigration to Finland – or being diagnosed before the age of 16 (*n* = 25), with nearly all individuals being foreign-born. To assess the robustness of our findings, we conducted a sensitivity analysis excluding these individuals. This exclusion did not substantially change the result, although reduced statistical power led to wider confidence intervals and higher p-values. Additionally, the difference between pre- and post-HIV diagnosis periods was slightly smaller compared to the main analysis.

## Discussion

To our knowledge, this is the first nationwide study to cover all PLWH and their diagnosed STIs over a 25-year period, both before and after HIV diagnosis. The use of national registers allowed for comprehensive inclusion of all PLWH in Finland, rather than a limited cohort. The reliability of the analyses was strengthened by obtaining dates of HIV and STIs from official records, rather than relying on self-reported data – except for a few immigrants diagnosed with HIV before coming to Finland.

One of the main findings was that a high proportion of individuals in the study population had no diagnosed STIs. Similar findings have been reported among cohorts of PLWH with shorter follow-up periods. In the Swiss cohort study (2017–2019) [2], 90% of participants were free of STIs, while in a U.S. cohort study from Washington, D.C. (2011–2015), the proportion was 93% [7, 9]. In contrast, only 41% (*n* = 151) of participants in a Brazilian study of women living with HIV (2014–2015), and 50% (*n* = 1961) in a U.S. Military Personnel HIV cohort study (2010–2018), were STI-free [14, 15]. These differences may be attributed to variations in age distributions – and thus differences in sexual behaviour – as well as to the different time periods during which the studies were conducted.

In our study, STI incidence increased over time. Since the year 2000, the incidence rate of STI events has been consistently higher before HIV diagnosis than after the diagnosis. Similarly, the extended Cox model showed that the predicted margins for hazard ratios of STIs after HIV diagnosis were equal to or lower than before the diagnosis across all transmission groups. Because all post-HIV STIs naturally occur later in calendar time than pre-HIV STIs, this temporal trend explains the slightly higher post-HIV overall STI incidence that was found only in the crude analysis where the time variable was not included. A similar trend was observed in a cohort of Italian MSM and a Malawian study assessing sexual behaviour following acute HIV diagnosis. Both studies found that although risk behaviours did not cease entirely, they tended to decrease after HIV diagnosis – for example, through reduced number of sexual partners and increased condom use [16, 17].

Among PLWH, MSM were identified as the group at highest risk for STIs. Although they represented approximately one-third of the study population, they accounted for more than half of the STI diagnostic events. MSM also had the highest STI diagnostic event incidence rate, both before and after HIV diagnosis. Compared to heterosexuals, MSM had a significantly higher risk of acquiring STIs – especially gonorrhoea, for which the risk was almost ten times greater. Similar patterns were observed in other studies. In the Swiss cohort study, MSM comprised 47% of the population but accounted for 88% of all STIs. Notably, MSM under the age of 50 made up only 21% of the cohort but were responsible for 61% of reported STIs [9]. Likewise, a U.S. cohort study in Washington, D.C., found particularly high STI rates among MSM and individuals aged 18–34 years. The overall STI incidence in that study was 1.2 times higher than in our cohort. Even more strikingly, a U.S. cohort study of MSM living in Birmingham, Alabama (2014–2019) reported STI incidence rates up to 20 times higher than those observed in our study [18]. The unexpectedly low incidence of STIs among PWID in our study contrasts with findings from the Swiss cohort study, where PWID were identified as being at higher risk for STIs [9]. Similarly, in a small Brazilian cohort study, the prevalence of STIs was 73% among women living with HIV who used drugs at their first visit to an STI clinic [14]. The low STI incidence among PWID in our cohort may reflect the characteristics of the HIV epidemic in this group, which has affected marginalized individuals who were older, had fewer sexual partners, and were less sexually active than PWID without HIV [19].

We found no significant increase in STI incidence immediately following the release of the Swiss Statement in 2008 [2]. However, the publication of the Partner1 study [3] and the U=U campaign in 2016 seems to coincide with the rise of STI incidences. This rise may, in part, reflect increased awareness of the minimal risk of HIV transmission, potentially leading to risk compensation and possibly increased testing. The high rates of syphilis after HIV diagnosis can be attributed to several factors. In Finland, routine syphilis testing is performed at many HIV clinics during annual follow-up visits, which may enhance case detection after HIV diagnosis. A substantial proportion of syphilis cases were identified among MSM, potentially due to serosorting – the practice of selecting sexual partners with the same HIV status. Additionally, syphilis incidence has increased at the population level over time, contributing to the observed trend. In this national PLWH cohort, 71% had no diagnosed STIs other than HIV. This highlights the continued importance of HIV testing whenever it is suspected or when diagnosing any HIV-related indicator condition – not limited to STIs alone.

STI testing and intensified counselling should particularly target individuals at the highest risk of testing positive, such as MSM and those with a history of STIs. Although regular testing can be part of routine HIV outpatient care, the limited frequency of these appointments highlights the need for accessible testing options in other healthcare or community settings. Counselling should emphasize the use of condoms in casual sexual relationships – even when the partner is on HIV PrEP or on ART – and include assessment of the individual’s suitability for doxycycline prophylaxis (doxy-PEP), in countries where it is available. When taken within 24–72 h after condomless sex, doxy-PEP has been shown to reduce the risk of bacterial STIs. It is effective in preventing chlamydia and syphilis among MSM living with HIV and MSM on HIV PrEP, while protection against gonorrhoea is limited or inconsistent due to widespread tetracycline resistance [20, 21].

STIs – particularly syphilis and gonorrhoea – are commonly diagnosed before HIV diagnosis, and individuals remain at elevated risk of acquiring HIV for several years following an STI [22–26]. Therefore, the occurrence of STIs prior to an HIV diagnosis represents a key opportunity for HIV prevention, including consideration of HIV PrEP. Our results suggest that STIs may be underdiagnosed among PLWH who inject drugs, highlighting the need for better counselling and testing approaches. HIV treatment centres serving PWID, among other services, could adopt a more initiative-taking role in addressing this issue.

One major limitation of this study is the absence of data on the total number of STI tests performed, which may introduce bias given that testing frequency and detection rates have likely increased over time. Diagnostic bias may also arise from differences in healthcare-seeking behaviour with MSM possibly more likely to undergo STI testing, while STI incidence among immigrants – particularly before HIV diagnosis – may be underestimated due to incomplete pre-migration health data, inconsistent PICs, and potential barriers to testing such as stigma, lack of information, and distrust of authorities. The absence of information on anatomical sampling sites, testing indications, sexual behaviour, and substance use constrained the assessment of behavioural risk factors. Anogenital herpes and human papillomavirus (HPV) infections were not included, as they are not reported in the NIDR. The binary gender classification in the registries restricted the ability to capture gender diversity. The absence of pre-HIV follow-up data for those diagnosed before 1995 or included in the cohort after HIV diagnosis, as well as the 50-year recurrence period for hepatitis C virus (HCV) in the NIDR, may have led to an underestimation of STI incidence, although sensitivity analyses excluding these individuals did not substantially affect the results.

In conclusion, the majority of PLWH had no recorded STIs other than HIV, underscoring the importance of HIV testing even in individuals without suspected or diagnosed STIs. STI risk remained stable or declined following HIV diagnosis, regardless of the HIV transmission mode, when adjusted for time-dependent variables. The STIs preceding HIV diagnosis highlight both the need and the opportunity to promote HIV PrEP. As STIs continue to occur after HIV diagnosis, individuals with a history of STIs – particularly MSM – should be offered regular testing, access to doxy-PEP, and condom promotion. Our findings also suggest that STIs among PWID may be underdiagnosed, warranting greater attention to STI testing within this group. Prevention and early diagnosis of STIs, including HIV, benefit both individuals and public health by interrupting transmission chains. These opportunities for prevention should not be overlooked.

## Supporting information

10.1017/S0950268826101150.sm001 Kaila et al. supplementary material 1 Kaila et al. supplementary material

10.1017/S0950268826101150.sm002 Kaila et al. supplementary material 2 Kaila et al. supplementary material

## Data Availability

The data that support the findings of this study are available on request for scientific research in anonymized form from Data Permit Authority Findata (https://www.findata.fi/en/ services/data-requests/). The data are not publicly available due to privacy or ethical restrictions.

## Supplementary material

The supplementary material for this article can be found at http://doi.org/10.1017/S0950268826101150.

## References

[1] Lundgren JD, et al. (2015) Initiation of antiretroviral therapy in early asymptomatic HIV infection. New England Journal of Medicine 373(9), 795–807. 10.1056/NEJMoa1506816. 26192873 PMC4569751

[2] Vernazza P, et al. (2008) Les personnes seropositives ne souffrant d’aucune autre MST et suivant un traitement antiretroviral efficace ne transmettent pas le VIH par voie sexuelle (HIV infected patients under HAART without any other sexually transmitted infection do not transmit HIV by sexual intercourse). Le Bulletin des Médecins de Suisses 89, 165–169.

[3] Rodger AJ, et al. (2016) Sexual activity without condoms and risk of HIV transmission in serodifferent couples when the HIV-positive partner is using suppressive antiretroviral therapy. Journal of American Medical Association 316(2), 171–181. 10.1001/jama.2016.5148. 27404185

[4] Rodger AJ, et al. (2019) Risk of HIV transmission through condomless sex in serodifferent gay couples with the HIV-positive PARTNER taking suppressive antiretroviral therapy (PARTNER): Final results of a multicentre, prospective, observational study. Lancet 393(10189), 2428–2438. 10.1016/S0140-6736(19)30418-0. 31056293 PMC6584382

[5] Broyles LN, et al. (2023) The risk of sexual transmission of HIV in individuals with low-level HIV viraemia: A systematic review. Lancet 402(10400), 464–471. 10.1016/S0140-6736(23)00877-2. 37490935 PMC10415671

[6] Shilaih M, et al. (2017) Factors associated with syphilis incidence in the HIV-infected in the era of highly active antiretrovirals. Medicine (Baltimore) 96(2), e5849. 10.1097/MD.0000000000005849. 28079818 PMC5266180

[7] Lucar J, et al. (2018) Sexually transmitted infections among HIV-infected individuals in the district of Columbia and estimated HIV transmission risk: Data from the DC cohort. Open Forum Infectious Diseases 5(2), ofy017. 10.1093/ofid/ofy017. 29479550 PMC5804762

[8] Secco AA, et al. (2020) Sexually transmitted infections in persons living with HIV infection and estimated HIV transmission risk: Trends over time from the DC cohort. Sexually Transmitted Infections 96(2), 89–95. 10.1136/sextrans-2019-054216. 31907326 PMC7031010

[9] Bosetti D, et al. (2022) Swiss HIV cohort study. Risk factors and incidence of sexually transmitted infections in the Swiss HIV cohort study. Open Forum Infectious Diseases 9(12), ofac592. 10.1093/ofid/ofac592. 36504700 PMC9728517

[10] Carias AM and Hope TJ (2019) Barriers of mucosal entry of HIV/SIV. Current Immunology Reviews 15(1), 4–13. 10.2174/1573395514666180604084404. 31853241 PMC6919325

[11] Brummer-Korvenkontio H et al. (2010) Recommendation National Institute for Health and Welfare (THL). https://urn.fi/URN:NBN:fi-fe201205085062

[12] HIV register. https://repo.thl.fi/sites/laaturekisterit/hiv-rekisteri/hivrekisteri_alue.html

[13] Mutru M, et al. (2022) Finnish HIV quality of care register (FINHIV). British Medical Journal Open 12(1), e053287. 10.1136/bmjopen-2021-053287. PMC8785158 35063958

[14] Tosato Boldrini NA, et al. (2021) Sexually transmitted infections among women living with HIV in a Brazilian city. Brazilian Journal of Infectious Diseases 25(1), 101044. 10.1016/j.bjid.2020.101044. PMC9392134 33417851

[15] Tzeng JS, et al. (2013) Epidemiology of sexually transmitted infections among human immunodeficiency virus positive United States military personnel. Journal of Sexually Transmitted Diseases 2013, 610258. 10.1155/2013/610258. 26316961 PMC4437416

[16] Camoni L, et al. (2011) Sexual behaviour reported by a sample of Italian MSM before and after HIV diagnosis. Annali dell’Istituto Superiore di Sanità 47(2), 214–219. 10.4415/ANN_11_02_14. 21709392

[17] Rucinski KB, et al. (2018) Sustained sexual behavior change after acute HIV diagnosis in Malawi. Sexually Transmitted Diseases 45(11), 741–746. 10.1097/OLQ.0000000000000873. 29870501 PMC6179914

[18] Gravett RM, et al. (2022) Bacterial sexually transmitted infection incidence among southern men who have sex with men with human immunodeficiency virus in the treatment as prevention era, 2014–2019. Clinical Infectious Diseases 75(8), 1446–1448. 10.1093/cid/ciac257. 35380640 PMC9555833

[19] Kivelä PS, et al. (2009) High prevalence of unprotected sex among Finnish HIV-positive and HIV-negative injecting drug users. Scandinavian Journal of Public Health 37(4), 357–363. 10.1177/1403494809105290. 19372233

[20] Molina JM, et al. (2018) Post-exposure prophylaxis with doxycycline to prevent sexually transmitted infections in men who have sex with men: An open-label randomised substudy of the ANRS IPERGAY trial. Lancet Infectious Diseases 18(3), 308–317. 10.1016/S1473-3099(17)30725-9. 29229440

[21] Luetkemeyer AF, et al. (2023) Postexposure doxycycline to prevent bacterial sexually transmitted infections. New England Journal of Medicine 388(14), 1296–1306. 10.1056/NEJMoa2211934. 37018493 PMC10140182

[22] Peterman TA, et al. (2015) Risk for HIV following a diagnosis of syphilis, gonorrhoea or chlamydia: 328,456 women in Florida, 2000–2011. International Journal of STD & AIDS 26(2), 113–119. 10.1177/0956462414531243. 24713228 PMC6755665

[23] Pathela P, et al. (2015) The high risk of an HIV diagnosis following a diagnosis of syphilis: A population-level analysis of New York City men. Clinical Infectious Diseases 15(2), 281–287. 10.1093/cid/civ289. 25870333

[24] Mallitt KA, et al. (2018) Identifying missed clinical opportunities for the earlier diagnosis of HIV in Australia, a retrospective cohort data linkage study. PLoS One 13(12), e0208323. 10.1371/journal.pone.0208323. 30521582 PMC6283600

[25] Tilchin C, et al. (2019) Human immunodeficiency virus diagnosis after a syphilis, gonorrhea, or repeat diagnosis among males including non-men who have sex with men: What is the incidence? Sexually Transmitted Diseases 46(4), 271–277. 10.1097/OLQ.0000000000000964. 30870326 PMC6426356

[26] Newman DR, et al. (2020) Rates of new human immunodeficiency virus (HIV) diagnoses after reported sexually transmitted infection in women in Louisiana, 2000–2015: Implications for HIV prevention. Clinical Infectious Diseases 70(6), 1115–1120. 10.1093/cid/ciz303. 30976788 PMC6790153

