# Supplementary figures and images for: Burden and risk factors of sexually transmitted infections before and after HIV diagnosis in a Finnish national HIV cohort, 1995–2019
Source: Epidemiol Infect. 2026 Feb 16;154:e37. doi: 10.1017/S0950268826101150 (PMC13100927; doi:10.1017/S0950268826101150)

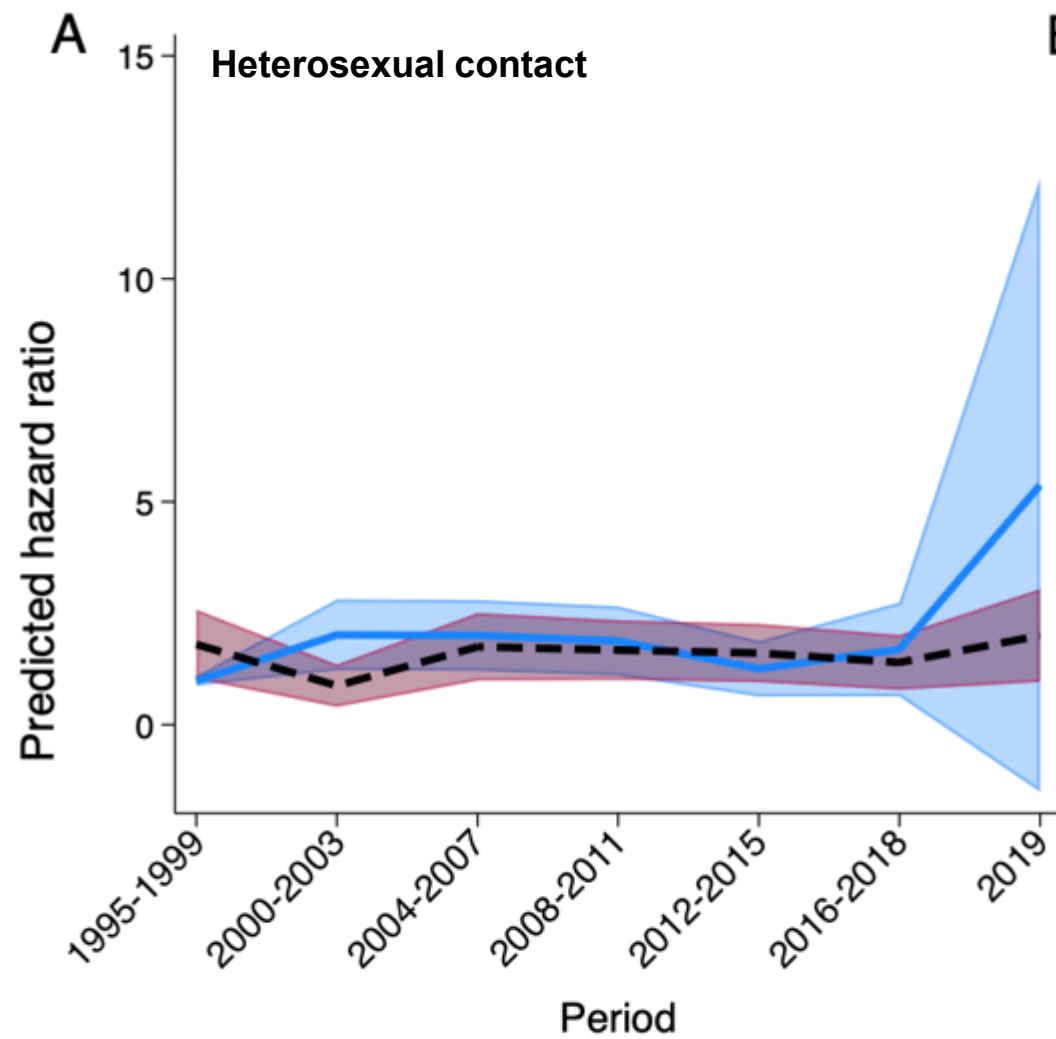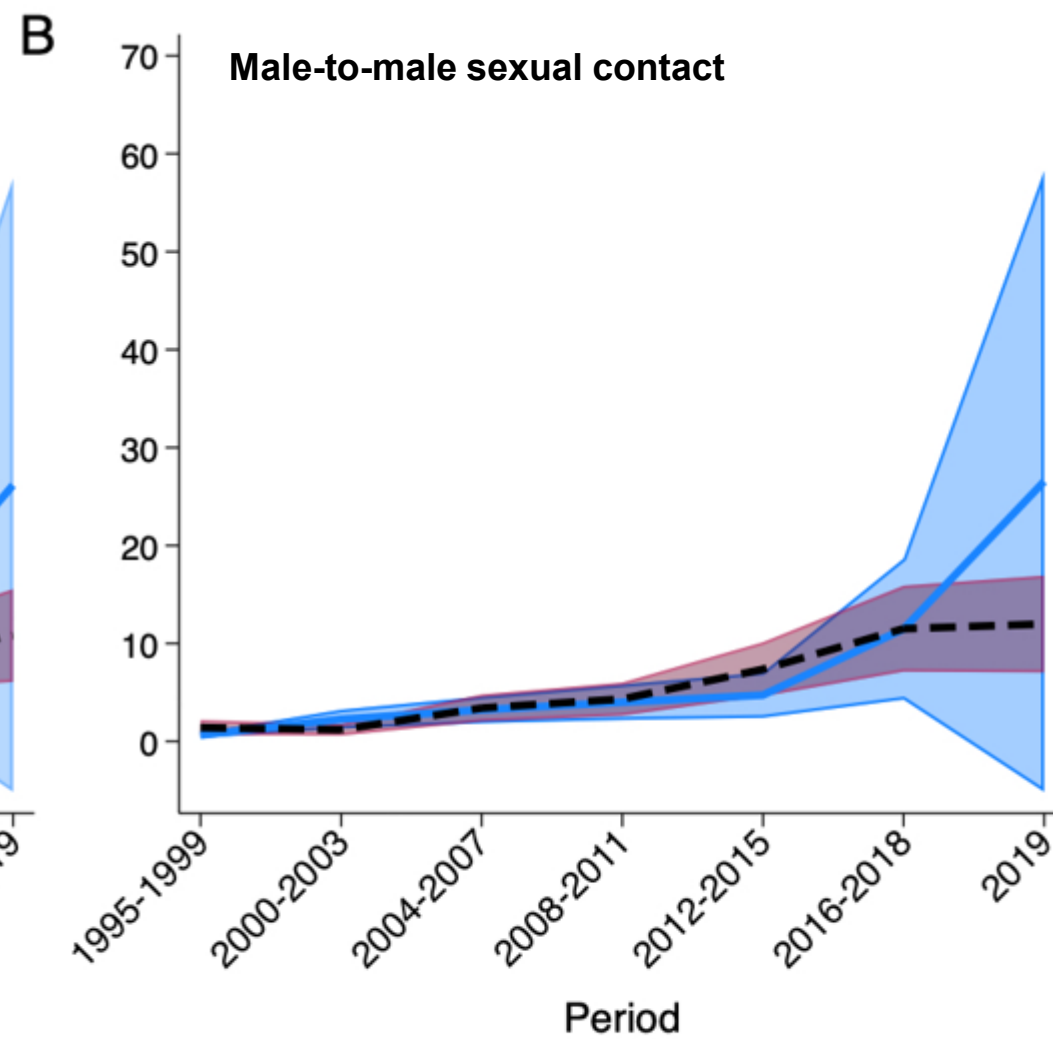

Before HIV-dg  
After HIV-dg

Supplement: Kaila et al. supplementary material 1 — Kaila et al. supplementary material [file S0950268826101150sup001.pdf]
