# Supplementary material for: Burden and risk factors of sexually transmitted infections before and after HIV diagnosis in a Finnish national HIV cohort, 1995–2019
Source: Epidemiol Infect. 2026 Feb 16;154:e37. doi: 10.1017/S0950268826101150 (PMC13100927; doi:10.1017/S0950268826101150)
Supplement: Kaila et al. supplementary material 2 — Kaila et al. supplementary material [file S0950268826101150sup002.docx]

**Predicted margins for hazard ratios for sexually transmitted infections by HIV transmission modes in the Finnish HIV Cohort, 1995‒2019: sensitivity analysis excluding STIs in the month prior to HIV diagnosis**

**All HIV Transmission Modes**

| **Time period** | **Relation to HIV diagnosis** | **Margin** | **Delta-method std. err.** | **z** | **P> IzI** | **[95%** | **CI]** |
| --- | --- | --- | --- | --- | --- | --- | --- |
| **1995–1999** | **Before** | 0.97049 | 0.0555653 | 17.47 | 0.000 | 0.8615841 | 1.079396 |
|  | **After** | 1.804388 | 0.3981123 | 4.53 | 0.000 | 1.024102 | 2.584674 |
| **2000–2003** | **Before** | 1.943554 | 0.3935269 | 4.94 | 0.000 | 1.172255 | 2.714852 |
|  | **After** | 0.8687177 | 0.2423617 | 3.58 | 0.000 | 0.3936974 | 1.343738 |
| **2004–2007** | **Before** | 1.981075 | 0.4016307 | 4.93 | 0.000 | 1.193893 | 2.768256 |
|  | **After** | 1.755632 | 0.3892432 | 4.51 | 0.000 | 0.9927297 | 2.518535 |
| **2008–2011** | **Before** | 1.88951 | 0.3979404 | 4.75 | 0.000 | 1.109562 | 2.669459 |
|  | **After** | 1.667731 | 0.3414952 | 4.88 | 0.000 | 0.998413 | 2.337049 |
| **2012–2015** | **Before** | 1.215336 | 0.3100674 | 3.92 | 0.000 | 0.6076153 | 1.823057 |
|  | **After** | 1.598302 | 0.3342651 | 4.78 | 0.000 | 0.9431542 | 2.253449 |
| **2016–2018** | **Before** | 1.74855 | 0.5471124 | 3.20 | 0.001 | 0.6762291 | 2.82087 |
|  | **After** | 1.425985 | 0.3193633 | 4.47 | 0.000 | 0.8000445 | 2.051926 |
| **2019** | **Before** | 5.294281 | 3.461587 | 1.53 | 0.126 | -1.490305 | 12.07887 |
|  | **After** | 2.00769 | 0.5340065 | 3.76 | 0.000 | 0.961057 | 3.054324 |

**Heterosexual contact**

| **Time period** | **Relation to HIV diagnosis** | **Margin** | **Delta-method std. err.** | **z** | **P> IzI** | **[95%** | **CI]** |
| --- | --- | --- | --- | --- | --- | --- | --- |
| **1995–1999** | **Before** | 0.9788925 | 0.0561143 | 17.44 | 0.000 | 0.8689105 | 1.088875 |
|  | **After** | 1.815855 | 0.4014085 | 4.52 | 0.000 | 1.029109 | 2.602601 |
| **2000–2003** | **Before** | 1.958235 | 0.3970134 | 4.93 | 0.000 | 1.180103 | 2.736367 |
|  | **After** | 0.8783548 | 0.2449466 | 3.59 | 0.000 | 0.3982683 | 1.358441 |
| **2004–2007** | **Before** | 2.026469 | 0.4091923 | 4.95 | 0.000 | 1.224467 | 2.828471 |
|  | **After** | 1.735067 | 0.3868952 | 4.48 | 0.000 | 0.9767663 | 2.493368 |
| **2008–2011** | **Before** | 1.868474 | 0.3957222 | 4.72 | 0.000 | 1.092873 | 2.644075 |
|  | **After** | 1.72436 | 0.3511878 | 4.91 | 0.000 | 1.036044 | 2.412675 |
| **2012–2015** | **Before** | 1.220848 | 0.3130068 | 3.90 | 0.000 | 0.607366 | 1.83433 |
|  | **After** | 1.610462 | 0.3368467 | 4.78 | 0.000 | 0.950255 | 2.27067 |
| **2016–2018** | **Before** | 1.725087 | 0.5490198 | 3.14 | 0.002 | 0.6490276 | 2.801145 |
|  | **After** | 1.439292 | 0.322827 | 4.46 | 0.000 | 0.8065624 | 2.072021 |
| **2019** | **Before** | 5.376717 | 3.512624 | 1.53 | 0.126 | -1.507899 | 12.26133 |
|  | **After** | 2.023365 | 0.5399544 | 3.75 | 0.000 | 0.965074 | 3.081656 |

**Male-to-male sexual contact**

| **Time period** | **Relation to HIV diagnosis** | **Margin** | **Delta-method std. err.** | **z** | **P> IzI** | **[95 %** | **CI]** |
| --- | --- | --- | --- | --- | --- | --- | --- |
| **1995–1999** | **Before** | 0.6279098 | 0.1471677 | 4.27 | 0.000 | 0.3394664 | 0.9163531 |
|  | **After** | 1.408455 | 0.4011962 | 3.51 | 0.000 | 0.6221246 | 2.194785 |
| **2000–2003** | **Before** | 2.207709 | 0.4878411 | 4.53 | 0.000 | 1.251558 | 3.16386 |
|  | **After** | 1.19742 | 0.3139782 | 3.81 | 0.000 | 0.5820337 | 1.812806 |
| **2004–2007** | **Before** | 3.341335 | 0.7169068 | 4.66 | 0.000 | 1.936223 | 4.746446 |
|  | **After** | 3.45936 | 0.7176178 | 4.82 | 0.000 | 2.052855 | 4.865865 |
| **2008–2011** | **Before** | 4.016585 | 0.9307459 | 4.32 | 0.000 | 2.192357 | 5.840814 |
|  | **After** | 4.482262 | 0.8955338 | 5.01 | 0.000 | 2.727048 | 6.237476 |
| **2012–2015** | **Before** | 4.584939 | 1.15246 | 3.98 | 0.000 | 2.32616 | 6.843719 |
|  | **After** | 7.313446 | 1.406756 | 5.20 | 0.000 | 4.556256 | 10.07064 |
| **2016–2018** | **Before** | 11.45895 | 3.678398 | 3.12 | 0.002 | 4.249426 | 18.66848 |
|  | **After** | 11.56065 | 2.254002 | 5.13 | 0.000 | 7.14289 | 15.97842 |
| **2019** | **Before** | 26.80313 | 16.32525 | 1.64 | 0.101 | -5.193775 | 58.80004 |
|  | **After** | 12.19669 | 2.569719 | 4.75 | 0.000 | 7.160138 | 17.23325 |

The same baseline reference category is used in all panels.
